# Diagnostic Concordance of CT and Ultrasound With Operative Findings in Acute Cholecystitis: A Retrospective Cohort Study

**DOI:** 10.7759/cureus.108267

**Published:** 2026-05-04

**Authors:** Thaddaeus R Castaneda, Amer A Afaneh

**Affiliations:** 1 Department of General Surgery, Mercy Health, St. Vincent Medical Center, Bon Secours Mercy Health, Toledo, USA

**Keywords:** acute cholecystitis, computed tomography, diagnostic concordance, gangrenous cholecystitis, ultrasound

## Abstract

Background

Acute cholecystitis (AC) is a common surgical emergency. Ultrasound (US) is the preferred initial imaging study, while CT is often used when findings are unclear. Reported imaging performance, however, varies. This study evaluated relationships among imaging, surgical, and pathological findings in patients who underwent cholecystectomy for acute disease.

Methods

This single-center retrospective review included 224 adults who underwent cholecystectomy between January 2020 and January 2025, with pathology-confirmed acute, acute-on-chronic, or gangrenous cholecystitis. Eligible patients received US, CT, or both within 48 hours before surgery. Imaging was considered positive if two or more inflammatory features were documented or if the radiologist’s impression indicated acute disease, including equivocal but concerning language. Operative and pathological findings served as reference standards. Sensitivity, concordance, and 95% CIs were calculated. Sensitivities were compared using two-proportion z-tests, McNemar’s test for paired data, and Cohen’s kappa (κ) for inter-modality agreement.

Results

CT showed higher sensitivity than US (126/176 (71.5%) vs 93/156 (59.6%); z = 2.30, p = 0.022) and similar concordance with operative findings. Among 108 patients who underwent both modalities, 24 (22.2%) had discordant results. CT was significantly more likely than US to agree with operative findings (McNemar’s χ² = 8.17, p = 0.004). Inter-modality agreement was moderate (κ = 0.53, 95% CI 0.37-0.69). Sensitivity in gangrenous cases was lower than overall sensitivity for both CT (15/29 (51.7%)) and US (6/24 (25.0%); z = 1.98, p = 0.048).

Conclusions

In this surgically managed cohort, CT and US provided complementary information. CT demonstrated better concordance with operative findings and modestly higher sensitivity, particularly in gangrenous cholecystitis, although the clinical significance remains uncertain. US remains the appropriate first-line imaging modality, with CT serving a complementary role in selected patients.

## Introduction

Background and clinical burden

Acute cholecystitis (AC) is one of the most common indications for emergency general surgery, accounting for an estimated 335,000 ED visits and more than three billion dollars in annual healthcare costs in the United States [1]. The condition typically reflects the progression of biliary colic to gallbladder inflammation following cystic duct obstruction, most often secondary to gallstones [2]. Although many cases present with a classic clinical picture, the accuracy of imaging interpretation and its alignment with operative findings can vary substantially across institutions, contributing to diagnostic uncertainty and delayed management.

Diagnostic criteria and severity scales

Standardized diagnostic frameworks have improved consistency in identifying and grading AC, but variation in real-world application remains significant. The Tokyo Guidelines 2018 (TG18) define AC based on a combination of local inflammatory signs, systemic inflammatory response, and imaging confirmation, offering a widely accepted, structured approach to diagnosis [3]. The World Society of Emergency Surgery (WSES 2020) guidelines expand this framework by emphasizing early recognition, risk assessment, and the optimal timing of laparoscopic intervention to reduce morbidity [4]. The American Association for the Surgery of Trauma (AAST) grading system complements diagnostic and imaging criteria by correlating operative findings with physiological severity, facilitating standardized comparisons across studies and clinical settings [5]. Despite these advances, diagnostic interpretation remains variable due to institutional protocols, radiologist expertise, and the heterogeneity of patient presentations, especially in patients with evolving inflammation or atypical features.

Imaging in AC

Ultrasound (US) and computed tomography (CT) remain the primary imaging modalities used in the diagnosis and evaluation of AC. Reported sensitivities vary widely, ranging from 60% to 90% for US and 70% to 90% for CT, depending on operator experience, timing, and underlying disease severity [6-9]. US is preferred as the first-line imaging study due to its accessibility and high accuracy for detecting gallstones. At the same time, CT provides more reliable visualization of gallbladder wall thickening, pericholecystic fluid, and pericholecystic fat stranding, especially in patients with complicated or gangrenous disease [10]. In clinical practice, discordance between imaging and the overall disease picture can contribute to uncertainty, repeat evaluations, and delays in surgical intervention. Interpretation may also vary based on institutional reporting practices and differences in how inflammatory features are emphasized across imaging reports. Clarifying how frequently such discrepancies occur and how they relate to disease severity is essential for improving diagnostic pathways and optimizing operative planning.

Study rationale and objectives

This study evaluated the concordance of US and CT findings with operative and pathological findings in patients undergoing cholecystectomy for AC. Secondary objectives included comparing the sensitivity of each imaging modality within this surgically managed cohort, assessing inter-modality agreement, and identifying clinical and laboratory factors associated with discordant imaging results. This retrospective cohort study has been reported in line with the STROCSS 2025 guidelines [11].

## Materials and methods

Study composition and setting

This single-center retrospective cohort study was conducted within a tertiary community hospital and a smaller affiliated community hospital in the same regional health system. Adults who underwent cholecystectomy for suspected acute gallbladder disease between January 2020 and January 2025 were included. Because retrospective operative cohorts are susceptible to selection bias and information bias, several measures were taken to mitigate these effects, including uniform inclusion and exclusion criteria, the use of finalized radiology reports from a single institutional group, and limiting imaging to a standardized 48-hour preoperative window. Because inclusion was limited to surgically managed patients with pathology-confirmed acute disease, this design is also subject to verification bias and may overestimate modality-specific sensitivity.

Eligibility criteria

**Figure 1:**
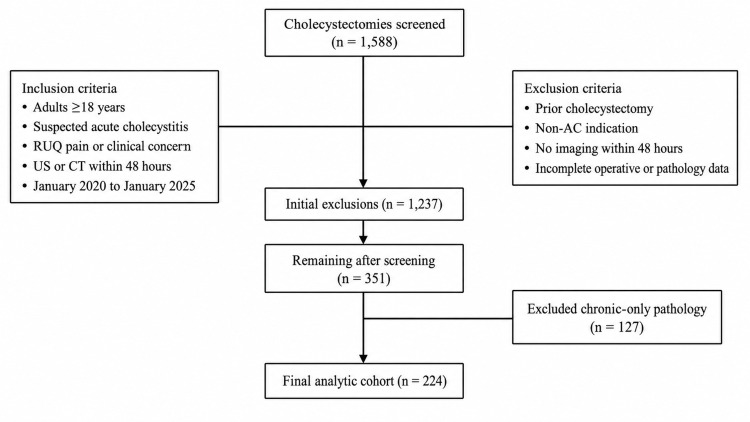
Study flow diagram.

A total of 1,588 cholecystectomy cases were screened for eligibility. Patients were included if they were 18 years or older, had pathology-confirmed acute, acute-on-chronic, or gangrenous cholecystitis, and underwent US, CT, or both within 48 hours of surgery. Exclusion criteria included chronic cholecystitis without acute inflammation, incomplete imaging or operative documentation, prior biliary surgery, and imaging performed outside the specified time window. After applying all criteria, 351 patients met initial eligibility based on qualifying preoperative imaging, and 127 with chronic-only pathology were excluded, resulting in a final analytic cohort of 224 patients. The screening process and final sample size are summarized in Figure 1.

Data collection

Demographic and clinical data collected from the electronic medical record included age, sex, race, presenting symptoms, symptom duration, white blood cell count, and comorbidities. Radiology reports were generated by the facility’s radiology group. Clinical notes were taken from the surgeon’s documentation. A trained investigator reviewed each finalized report and recorded all predefined imaging variables in a standardized data collection spreadsheet, including gallbladder wall thickening, pericholecystic fluid, gallstones, wall hyperenhancement, pericholecystic stranding, and, for US, the sonographic Murphy sign. Operative notes were reviewed for wall thickening, pericholecystic fluid, gangrenous changes, perforation, and intraoperative complications. Pathology reports were used to confirm acute inflammation, acute-on-chronic changes, necrosis, gangrene, hemorrhage, microabscesses, and gallstones.

Imaging definitions

Imaging was considered positive when two or more predefined inflammatory features were documented in the finalized report and/or when the radiologist’s impression favored AC, acute-on-chronic cholecystitis, or gangrenous cholecystitis, in accordance with established diagnostic frameworks [3,4]. Equivocal impression language, including phrases such as “possible,” “cannot exclude,” or “correlate clinically,” was coded as positive when the report was interpreted as concerning for acute gallbladder inflammation. Imaging classification was based on the finalized report content rather than on blinded re-review of images to reflect real-world clinical interpretation. US served as the initial imaging modality per institutional workflow, while CT was commonly performed in patients with atypical symptoms or suspected complicated disease.

Outcome measures

The primary outcome was concordance of US and CT with operative and pathological reference standards. Secondary outcomes included modality-specific sensitivity within this surgically managed cohort, inter-modality agreement, and associations between discordant imaging results and clinical or pathological characteristics. Sensitivity reflected whether each modality identified any AC in pathology-confirmed cases, whereas concordance represented agreement between the imaging severity classification and the operative or pathological severity category. A modality could therefore demonstrate sensitivity without concordance if it correctly detected acute disease but underestimated its severity.

Statistical analysis

Continuous variables were summarized as means with SDs or medians with IQRs, and categorical variables were summarized as frequencies and percentages. Sensitivity and 95% CIs were calculated for each imaging modality, with operative and pathological findings as the reference standards. CIs for proportions were calculated using the Wilson score method with a free online calculator (https://www.statskingdom.com/proportion-confidence-interval-calculator.html, accessed November 5, 2025).

Comparisons of sensitivity between CT and US were performed using two-proportion z-tests (two-sided, α = 0.05). Two-proportion z-tests were also used to compare concordance proportions across disease severity subgroups. Among patients who underwent both imaging modalities, McNemar’s test was used to assess paired concordance with operative findings. Cohen’s kappa (κ) statistic was calculated to evaluate inter-modality agreement.

Because all patients in the analytic cohort had pathology-confirmed acute disease, specificity, positive predictive value, negative predictive value, and overall diagnostic accuracy could not be estimated. Accordingly, sensitivity estimates should be interpreted within the context of a selected operative cohort and may not be generalizable to all patients evaluated for suspected AC. Statistical significance was defined as p < 0.05. Data management and analysis were performed using SAS version 9.4 (SAS Institute, Cary, NC, USA).

Ethical considerations

The institutional review board approved this study. Because it involved a retrospective review of de-identified medical records, informed consent was not required. All data were handled in accordance with institutional and federal standards for the protection of human subjects.

## Results

A total of 224 patients met the inclusion criteria for the final analytic cohort after all eligibility filters and exclusions were applied. These patients represented the operative subset with pathology-confirmed acute, acute-on-chronic, or gangrenous cholecystitis who also underwent qualifying preoperative imaging within 48 hours of surgery. Figure 1 summarizes the overall screening process and final sample size.

Cohort characteristics

The demographic and clinical characteristics are summarized in Table 1. The mean age was 52.4 years, and 128 (57.1%) patients were female. Patients with gangrenous cholecystitis were older than those with acute or acute-on-chronic disease (mean, 60.7 vs. 50.8 years). WBC counts were also higher in gangrenous cases (16.9 × 10³/µL vs. 11.0 × 10³/µL). Hypertension was more common among patients with gangrenous disease than among those with acute or acute-on-chronic cholecystitis (22/36 (61.1%) vs. 76/188 (40.4%)), as was diabetes (11/36 (30.6%) vs. 32/188 (17.0%)). Presenting symptoms, including right upper quadrant pain, fever, and nausea or vomiting, were similar across groups.

**Table 1:** Demographic and clinical presentation. RUQ: Right upper quadrant.

Variable	All subjects (n = 224)	Acute/acute-on-chronic (n = 188)	Gangrenous (n = 36)
Age, mean (SD)	52.4 (18.3)	50.8 (18.0)	60.7 (18.1)
Sex, n (%)			
Female	128 (57.1)	114 (60.6)	14 (38.9)
Male	96 (42.9)	74 (39.4)	22 (61.1)
Race/ethnicity, n (%)			
White	182 (81.3)	154 (81.9)	28 (77.8)
Black or African American	28 (12.5)	24 (12.8)	4 (11.1)
Hispanic	9 (4.0)	6 (3.2)	3 (8.3)
Asian	1 (0.5)	1 (0.5)	0 (0)
Other	4 (1.8)	3 (1.6)	1 (2.8)
RUQ pain, n (%)	218 (97.3)	183 (97.3)	35 (97.2)
Nausea/vomiting, n (%)	139 (62.1)	117 (62.2)	22 (61.1)
Fever, n (%)	14 (6.3)	11 (5.9)	3 (8.3)
Duration of symptoms, median (IQR)	2.0 (1.0, 3.0)	2.0 (1.0, 3.0)	2.0 (1.0, 2.5)
Murphy’s sign positive, n (%)	36/104 (34.5)	29/87 (33.3)	7/17 (41.2)
Abdominal tenderness, n (%)	127 (56.7)	106 (56.4)	21 (58.3)
Rebound tenderness, n (%)	7 (3.1)	5 (2.7)	2 (5.6)
WBC count, mean (SD)	12.0 (5.6)	11.0 (4.6)	16.9 (7.9)
Diabetes, n (%)	43 (19.2)	32 (17.0)	11 (30.6)
Hypertension, n (%)	98 (43.8)	76 (40.4)	22 (61.1)
Previous biliary surgery, n (%)	0 (0)	0 (0)	0 (0)

Imaging findings

Imaging findings are summarized in Table 2. A total of 156 patients underwent US, 176 underwent CT, and 108 received both modalities within 48 hours before surgery. Across all US studies, gallstones were identified in 127 (81.4%), gallbladder wall thickening in 91 (58.3%), and pericholecystic fluid in 57 (36.5%). CT demonstrated gallbladder wall thickening in 112 (63.6%), pericholecystic fluid in 105 (59.7%), and gallstones in 139 (79.0%) studies. Median wall thickness measured 5 mm on US and 10 mm on CT, although numeric wall measurements were explicitly reported in only 50 US examinations and five CT examinations. Patients with gangrenous cholecystitis demonstrated higher CT rates of wall thickening than those with acute or acute-on-chronic disease (21/29 (72.4%) vs. 91/147 (61.9%)) and higher rates of pericholecystic stranding (8/29 (27.6%) vs. 29/147 (19.7%)).

**Table 2:** Imaging findings. ^a ^Wall measurement was available for 50 US examinations. ^b^ Wall measurement was available for five CT examinations. GB: Gallbladder.

Variable	All subjects (n = 224)	Acute/acute-on-chronic (n = 188)	Gangrenous (n = 36)
A. Ultrasound findings (n = 156)			
GB wall thickening, n (%)	91 (58.3)	78 (59.1)	13 (54.2)
GB wall measurement, median mm (IQR)^a	5.0 (4.0, 6.8)	4.9 (4.0, 6.8)	5.0 (4.3, 6.0)
Pericholecystic fluid, n (%)	57 (36.5)	52 (39.4)	5 (20.8)
Gallstones, n (%)	127 (81.4)	110 (83.3)	17 (70.8)
Sonographic Murphy’s sign positive, n (%)	15/125 (12.0)	13/108 (12.0)	2/17 (11.8)
B. CT findings (n = 176)			
CT GB wall thickening, n (%)	112 (63.6)	91 (61.9)	21 (72.4)
GB wall measurement, median mm (IQR)^b	10.0 (9.0, 10.0)	10.0 (9.0, 10.0)	–
CT pericholecystic fluid, n (%)	105 (59.7)	87 (59.2)	18 (62.1)
CT gallstones, n (%)			
Yes	139 (79.0)	116 (78.9)	23 (79.3)
No	34 (19.3)	28 (19.1)	6 (20.7)
Not visualized	3 (1.7)	3 (2.0)	0 (0)
Distended GB, n (%)	80 (45.5)	70 (47.6)	10 (34.5)
Hyperenhancement, n (%)	6 (3.4)	5 (3.4)	1 (3.5)
Pericholecystic stranding, n (%)	37 (21.0)	29 (19.7)	8 (27.6)

Operative and pathological findings

Operative and pathological findings are summarized in Table 3. Intraoperative assessment commonly demonstrated gallbladder wall thickening in 201 (89.7%) patients and pericholecystic fluid in 200 (89.3%). Gangrenous changes were identified intraoperatively in 39 (17.4%) patients and confirmed on pathology in 43 (19.2%) specimens, reflecting the greater sensitivity of histologic evaluation for detecting early or localized necrosis that may not be visible during laparoscopy. Necrosis was substantially more frequent in gangrenous cases than in acute or acute-on-chronic disease (25/36 (69.4%) vs. 23/188 (12.2%)), while hemorrhage remained uncommon across groups. Gallstones were present in 206 (92.0%) specimens. Only one intraoperative complication occurred, and no intraoperative mortalities were reported.

**Table 3:** Operative and pathological findings. *Veress needle liver puncture and subsequent limited visualization were the reasons for conversion. GB: Gallbladder.

Variable	All subjects (n = 224)	Acute/acute-on-chronic (n = 188)	Gangrenous (n = 36)
A. Operative findings			
Surgery type, n (%)			
Laparoscopic	219 (97.8)	185 (98.4)	34 (94.4)
Converted	5 (2.2)	3 (1.6)	2 (5.6)
Intraoperative GB wall thickening, n (%)	201 (89.7)	165 (87.8)	36 (100)
Intraoperative pericholecystic fluid, n (%)	200 (89.3)	167 (88.8)	33 (91.7)
Gangrenous cholecystitis, n (%)	39 (17.4)	3 (1.6)	36 (100)
Emphysematous changes, n (%)	2 (0.9)	1 (0.5)	1 (2.8)
Perforation, n (%)	9 (4.0)	6 (3.2)	3 (8.3)
Bile leakage, n (%)	11 (4.9)	9 (4.8)	2 (5.6)
Surgical complications*, n (%)	1 (0.5)	1 (0.5)	0 (0)
B. Pathology findings			
Wall inflammation, n (%)	206 (92.0)	170 (90.4)	36 (100)
Gangrene, n (%)	43 (19.2)	7 (3.7)	36 (100)
Necrosis, n (%)	48 (21.4)	23 (12.2)	25 (69.4)
Hemorrhage, n (%)	11 (4.9)	10 (5.3)	1 (2.8)
Gallstones in specimen, n (%)	206 (92.0)	174 (92.6)	32 (88.9)
Microabscesses, n (%)	7 (3.1)	5 (2.7)	2 (5.6)

Diagnostic concordance and sensitivity

Diagnostic concordance with operative and pathological findings is summarized in Table 4. In acute and acute-on-chronic disease, CT demonstrated higher concordance than US for both operative findings (111/147 (75.5%) vs. 87/132 (65.9%)) and pathological findings (111/147 (75.5%) vs. 86/132 (65.2%)). Concordance was lower in gangrenous cases but again favored CT for both operative findings (15/29 (51.7%) vs. 6/24 (25.0%)) and pathological findings (15/29 (51.7%) vs. 6/23 (26.1%)). All concordance comparisons were statistically significant by two-proportion z-tests.

**Table 4:** Concordance with operative and pathological findings. Comparisons between acute/acute-on-chronic and gangrenous concordance proportions were performed using two-proportion z-tests; the corresponding test statistics and p-values are reported in the table. AOC: Acute-on-chronic.

Comparison	Acute/AOC, n/N (%)	95% CI	Gangrenous, n/N (%)	95% CI
CT vs. operative findings (z = 2.60, p = 0.009)	111/147 (75.5)	(68.0, 81.8)	15/29 (51.7)	(34.4, 68.6)
Ultrasound vs. operative findings (z = 3.76, p < 0.001)	87/132 (65.9)	(57.5, 73.4)	6/24 (25.0)	(12.0, 44.9)
CT vs. pathological findings (z = 2.60, p = 0.009)	111/147 (75.5)	(68.0, 81.8)	15/29 (51.7)	(34.4, 68.6)
Ultrasound vs. pathological findings (z = 3.52, p < 0.001)	86/132 (65.2)	(56.7, 72.7)	6/23 (26.1)	(12.5, 46.5)

Sensitivity results are presented in Table 5. Overall sensitivity was higher for CT than for US (126/176 (71.5%) vs. 93/156 (59.6%); z = 2.30, p = 0.022). A similar pattern was observed in acute and acute-on-chronic disease (111/147 (75.5%) vs. 87/132 (65.9%); z = 1.76, p = 0.078). CT also demonstrated greater sensitivity in gangrenous cholecystitis (15/29 (51.7%) vs. 6/24 (25.0%); z = 1.98, p = 0.048).

**Table 5:** Sensitivity analysis of CT and ultrasound. Two-proportion z-tests were used to compare sensitivity between CT and ultrasound. Statistical significance was defined as p < 0.05. AOC: Acute-on-chronic.

Subtype	CT sensitivity	95% CI	US sensitivity	95% CI	z	p-value
All subjects	126/176 (71.5%)	(64.5, 77.8)	93/156 (59.6%)	(51.8, 67.0)	2.3	0.022*
Acute/AOC	111/147 (75.5%)	(68.0, 81.8)	87/132 (65.9%)	(57.5, 73.4)	1.76	0.078
Gangrenous	15/29 (51.7%)	(34.4, 68.6)	6/24 (25.0%)	(12.0, 44.9)	1.98	0.048*

Paired imaging agreement

Paired imaging findings for the 108 patients who underwent both modalities are summarized in Table 6. Overall, CT and US agreed in 84 (77.8%) patients and disagreed in 24 (22.2%). In discordant cases, CT was concordant with operative findings in 19 patients, whereas US was concordant in only five, yielding a significant difference under McNemar’s test (χ² = 8.17, p = 0.004). Inter-modality agreement was moderate (κ = 0.53, 95% CI 0.37-0.69), indicating that CT and US provide overlapping but not fully interchangeable information in the evaluation of acute gallbladder disease.

**Table 6:** Paired CT and ultrasound concordance with operative findings. McNemar’s χ² = 8.17, p = 0.004 for paired CT and ultrasound comparisons. Cohen’s κ = 0.53 (95% CI, 0.37 to 0.69), indicating moderate agreement. ^†^Discordant indicates that one modality was concordant with operative findings while the other was not.

CT/US	US concordant with operative findings	US not concordant with operative findings	Total CT
CT concordant with operative findings	57	19†	76
CT not concordant with operative findings	5†	27	32
Total US	62	46	108

Clinical characteristics of discordant cases

Clinical and pathological characteristics of discordant cases are summarized in Table 7. Discordant imaging was associated with a higher rate of gangrenous cholecystitis on pathology (7/24 (29.2%) vs. 10/84 (11.9%)) and slightly lower rates of acute-on-chronic inflammation (14/24 (58.3%) vs. 57/84 (67.9%)). These patterns suggest that patients with more advanced or evolving inflammatory changes were more likely to have discordant imaging.

**Table 7:** Pathological outcomes by imaging concordance. Values are presented as n (%).

Outcome	US and CT agreed (n = 84)	US and CT disagreed (n = 24)
Acute, n (%)	17 (20.2)	3 (12.5)
Acute-on-chronic, n (%)	57 (67.9)	14 (58.3)
Gangrenous, n (%)	10 (11.9)	7 (29.2)

## Discussion

Principal findings

In this five-year retrospective cohort study of 224 patients diagnosed with acute, acute-on-chronic, or gangrenous cholecystitis, the primary finding was that CT showed closer alignment with operative and pathological findings than US, particularly in cases of more advanced disease. Within this surgically managed cohort, CT also showed modestly higher sensitivity. Inter-modality agreement was limited, with a significant discrepancy in one out of five patients, and discordant cases more frequently exhibited elevated white blood cell counts and gangrenous pathology. These findings suggest that disagreement between modalities may reflect variability in inflammatory progression and disease severity within this operative cohort.

Comparison with prior studies

The sensitivities observed in this study (126/176 (71.5%) for CT and 93/156 (59.6%) for US) were slightly lower than the 80%-90% ranges commonly reported in larger meta-analyses [6,8]. These differences likely reflect real-world variation in radiologist interpretation, institutional reporting practices, and the inclusion of all acute severities, including gangrenous cases, rather than a focus only on uncomplicated disease. Consistent with findings from Fagenholz PJ et al. [9] and Yeh DD et al. [10], CT demonstrated greater sensitivity in detecting gangrenous changes, whereas US remained highly reliable in identifying gallstones. Together, these patterns reinforce the complementary roles of US and CT in the diagnostic evaluation of acute cholecystitis. However, because the present cohort included only patients who underwent cholecystectomy for pathology-confirmed acute disease, these sensitivity estimates should not be interpreted as equivalent to overall diagnostic performance in a broader emergency or outpatient population.

Clinical interpretation

These results emphasize the importance of combining clinical evaluation with imaging findings. Right upper quadrant pain, fever, and nausea were common presenting symptoms, but they did not reliably distinguish gangrenous from non-gangrenous disease. CT aligned more closely with operative findings in cases of advanced inflammation, supporting its complementary value when the clinical picture suggests complicated disease. When imaging findings conflict with the clinical picture, features such as an elevated white blood cell count and persistent symptoms may support obtaining cross-sectional imaging. For patients with inconclusive, incomplete, or equivocal US reports but ongoing clinical concerns, early CT may help detect complicated disease and support timely surgical intervention. At the same time, US remains the appropriate first-line imaging modality because of its accessibility and established role in initial evaluation.

Strengths and limitations

This study has several strengths, including a consecutive five-year cohort, a standardized 48-hour imaging window, and the use of operative and pathological findings as reference standards. The paired-imaging subgroup enabled a direct comparison of US and CT performance within the same patients, thereby strengthening internal validity.

Several limitations should also be noted. Because only operative cases with pathology-confirmed acute disease were included, this study is subject to verification bias. As a result, specificity, positive predictive value, negative predictive value, and overall diagnostic accuracy could not be evaluated, and modality-specific sensitivity may be overestimated. The findings are therefore not generalizable to all patients evaluated for suspected acute cholecystitis.

Imaging classification was based on finalized radiology reports rather than blinded image re-review. Although this approach was intended to reflect the information available to the treating surgical team, it may have introduced reporting bias if secondary inflammatory signs were not fully itemized in the report. This limitation is particularly relevant to Murphy’s sign, which was likely underdocumented in both surgeon and radiology documentation. In some cases, prior emergency department pain treatment may also have reduced the ability to elicit or document the sign; therefore, the observed frequency should not be interpreted as its true prevalence in the cohort.

The observed difference in median gallbladder wall thickness between US and CT should also be interpreted cautiously because numeric wall measurements were explicitly reported in only 50 US studies and five CT studies. In addition, CT assessment of wall thickness may be influenced by edema, adjacent inflammatory change, and spatial resolution-related partial volume effects. Variation in radiologist interpretation and reporting practices may have influenced imaging classification overall, and interpretation by a single radiology group may limit generalizability to other settings. Differences in the timing of imaging within the 48-hour window may have affected the detection of evolving inflammatory changes. In addition, potentially relevant confounders, including disease severity and comorbidity burden, were not assessed in multivariable analysis. Finally, images were not re-reviewed in a blinded fashion, which prevented assessment of inter-reader variability. These factors should be considered when applying the findings to broader clinical practice.

Future directions

Future work should evaluate structured reporting templates for US and CT to improve interpretive consistency and reduce variation in report style or clinical context. Prospective studies incorporating blinded image review or dual-reader interpretation may better quantify inter-radiologist variability, clarify the role of equivocal report language, and strengthen diagnostic reproducibility. Additional research focused on the timing of imaging, particularly early versus late acquisition within the disease course, may clarify how inflammatory progression influences modality performance. Multicenter studies that include both operative and non-operative patients would allow estimation of specificity and yield more generalizable accuracy estimates. Finally, collaborative case review between radiology and surgery may help identify imaging features that correlate most reliably with operative severity and guide future reporting standards.

## Conclusions

CT and US each provide complementary information in the evaluation of acute gallbladder disease. In this surgically managed cohort, CT demonstrated better concordance with operative and pathological severity and modestly higher sensitivity, particularly in gangrenous cholecystitis. US remains the appropriate first-line imaging modality, with CT serving a complementary role when clinical concern persists after initial ultrasound or when disease severity is uncertain.
